# Electrophysiological Insights into Antibiotic Translocation and Resistance: The Impact of Outer Membrane Proteins

**DOI:** 10.3390/membranes14070161

**Published:** 2024-07-20

**Authors:** Ishan Ghai

**Affiliations:** Department of Life Sciences and Chemistry, Jacobs University Bremen, 28719 Bremen, Germany; isghai@jacobs-university.de

**Keywords:** antibiotic resistance, Gram-negative bacteria, outer membrane permeability, protein channels, molecular mechanisms, single-channel conductance, electrophysiological reversal potential

## Abstract

The alarming rise of antibiotic resistance in Gram-negative bacteria has emerged as a major global health challenge. A key factor contributing to this crisis is the low permeability of the bacterial outer membrane, which acts as a barrier that prevents antibiotics from entering the cell. Protein channels embedded in this outer membrane selectively regulate the influx of hydrophilic compounds, including antibiotics. To combat antibiotic resistance, understanding the molecular mechanisms governing antibiotic permeability through bacterial membrane channels is crucial. This knowledge is key towards elucidating their roles in studing antibiotic resistance. By compiling and analysing the flux data from multiple electrophysiological reversal potential experimental studies, which involves measuring zero-current potentials and the corresponding single-channel conductance, we can calculate the flux of charged antibiotics/compounds across different Gram-negative bacterial outer membrane channels. Through this comprehensive synthesis, this review aims to advance our understanding and stimulate discussions about the physicochemical factors influencing the flux of antibiotics through bacterial membrane protein channels, ultimately enhancing our knowledge in this area.

## 1. Introduction

The escalating threat of antibiotic resistance has outpaced the development of new antibiotics, creating an urgent global health crisis [1,2,3]. Many existing antibiotics lack effectiveness in managing the most resistant pathogens, such as *Klebsiella pneumoniae*, *Staphylococcus aureus*, *Klebsiella pneumoniae*, *Neisseria gonorrhoea*, *Escherichia coli*, and more existing and upcoming smart bacterial strains [3,4,5,6,7,8,9,10,11]. The challenge of antibiotic development, both scientifically and clinically, has led large pharmaceutical companies to shift their focus to more lucrative endeavours such as chronic diseases and lifestyle medications [12,13,14,15]. This shift is primarily because of the high cost and complex nature of antibiotic research, compounded by stringent regulatory requirements and the relatively lower return on investment compared to other drug categories [12,13,16,17,18,19]. Small and mid-sized companies with limited resources are often left to undertake the extensive research and development required for new antibiotics [17,18,19].

This situation is particularly troubling with regard to Gram-negative bacteria, where *E. coli*, *K. pneumoniae*, and *P. aeruginosa* have developed sophisticated mechanisms to evade existing antibiotics, making infections increasingly difficult to treat [20,21,22,23]. The clinical impact is severe, as these resistant infections lead to higher morbidity, mortality, and healthcare costs [21,22]. The lack of novel treatments exacerbates the risk of untreatable infections, highlighting a critical gap in our current healthcare system’s ability to combat bacterial resistance [18,19,20,21,22].

## 2. Understanding the Outer Barrier

Gram-negative bacteria are notorious for causing significant harm to humans, making them one of the most formidable threats [21,24,25,26]. These bacteria possess a complex cell envelope with an outer membrane that acts as a molecular sieve, creating a selective barrier to permeation [26,27]. This outer membrane primarily consists of a lipid bilayer that is impermeable to hydrophilic molecules [26,28]. The regulation of influx amongst these molecules are primarily controlled by specific membrane proteins [24,26,28,29], also known as porins (OM-Ps), a type of outer membrane channel. 

These OM-Ps shows a variety of structures, substrate specificities, regulatory mechanisms, and expressions [28]. Typically, they form hollow beta-barrel structures with a hydrophobic exterior. See Table 1 for different identified OM-Ps. The beta-barrel structure creates a transmembrane pore that allows passive diffusion of hydrophilic substances [26,28]. In addition, these proteins can be ion-selective, favouring either cations or anions [30,31]. Bacteria can change these proteins to reduce or inhibit influx, which, along with efflux systems, may contribute to antibiotic resistance [32,33]. 

Understanding membrane permeability at the molecular level is a critical task for drug design, as it plays a significant role in bacterial resistance processes [32,34]. Antibiotics or similar compounds such as small beta-lactamase inhibitors, penicillin, carbapenems, cephalosporins, and fluoroquinolones are assumed to enter Gram-negative bacteria through these OM-Ps. Even minor mutations in these OM-Ps may significantly affect antibiotic efficacy [10,34,35,36,37,38,39,40].

**Table 1:** This table presents approximate turnover rates (molecules/second) at specified concentration gradients for various OM-Ps with different substrates, updating the author’s prior work [14]. For precise calculations and methodologies, please refer to the cited research publications [40,41,42,43,44,45,46,47,48]. The columns in the table include substrates, reported OM-Ps, Gram-negative bacterial species, recalculated flux rates for comparative purposes only, approximate values, and reported flux rates (molecules/second) at specific gradients. While the author has made every effort to ensure the accuracy of the data and citations, the possibility of errors cannot be ruled out. This table aims to be thorough but may not include all existing data. Values are obtained from the literature.

Class	Molecules	Molecular Weight g·mol^−1^	Porins	Species	Recalculated Flux (Molecules/Second) at 1 μM Antibiotic Gradient	Reported Flux Molecules/Second at the Specific Mentioned Gradient
β-lactamase inhibitor	Avibactam	265.24	OmpF	*E. coli*	62	≈620 at gradient 10 μM [44]
β-lactamase inhibitor	Avibactam	265.24	OmpC	*E. coli*	23	≈229 at gradient 10 μM [45]
β-lactamase inhibitor	Tazobactam	300.29	OmpF	*E. coli*	20	≈200 at gradient 10 μM [44]
β-lactamase inhibitor	Tazobactam	300.29	OmpC	*E. coli*	20	≈200 at gradient 10 μM [45]
β-lactamase inhibitor	Sulbactam	233.24	OmpF	*E. coli*	20	≈200 at gradient 10 μM [44]
β-lactamase inhibitor	Sulbactam	233.24	OmpC	*E. coli*	18	≈187 at gradient 10 μM [45]
β-lactamase inhibitor	Sulbactam	233.24	DcaP	*A. baumannii*	8	≈8 at gradient 1 μM [48]
Phosphonic antibiotic	Fosfomycin	138.059	OprO	*P. aeruginosa*	28	≈280 at gradient 10 μM [42]
Phosphonic antibiotic	Fosfomycin	138.059	OprP	*P. aeruginosa*	≤1	≈2.2 at gradient 10 μM [42]
Phosphonic antibiotic	Fosfomycin	138.059	OmpF	*E. coli*	37	≈37 at gradient 1 μM [41]
Phosphonic antibiotic	Fosfomycin	138.059	OmpC	*E. coli*	7	≈7 at gradient 1 μM [41]
Phosphonic antibiotic	Fosfomycin	138.059	LamB	*E. coli*	3	≈3 at gradient 1 μM [41]
Phosphonic antibiotic	Fosfomycin	138.059	PhoE	*E. coli*	9	≈9 at gradient 1 μM [41]
Cephalosporin	Ceftazidime	546.57	OmpF	*E. coli*	34	≈1000 at gradient 30 μM [40]
Cephalosporin	Ceftazidime	546.57	OmpC	*E. coli*	17	≈500 at gradient 30 μM [40]
Cephalosporin	Ceftazidime	546.57	OprE	*P. aeruginosa*	≤1	≈0.4 at gradient 10 μM [47]
Cephalosporin	Cefotaxime	455.46	OprE	*P. aeruginosa*	≤1	≈0.1 at gradient 10 μM [47]
Penicillin	Ampicillin	349.41	OmpF	*E. coli*	24	≈237 at gradient 10 μM [43]
Penicillin	Benzylpenicillin	334.39	OmpF	*E. coli*	12	≈120 at gradient 10 μM [43]
Penicillin	Carbenicillin	378.40	OprE	*P. aeruginosa*	≤1	≈0.04 at gradient 10 μM [47]
Aminoglycoside	Gentamicin	477.60	OmpF	*E. coli*	1.5	≈15 at gradient 10 μM [46]
Aminoglycoside	Gentamicin	477.60	OmpC	*E. coli*	≤1	≈8 at gradient 10 μM [46]
Aminoglycoside	Gentamicin	477.60	LamB	*E. coli*	≤1	≤1 at gradient 10 μM [46]
Aminoglycoside	Gentamicin	477.60	Chip	*E. coli*	≤1	≈3 at gradient 10 μM [46]
Aminoglycoside	Kanamycin	484.50	OmpF	*E. coli*	1	≈10 at gradient 10 μM [46]
Aminoglycoside	Kanamycin	484.50	OmpC	*E. coli*	1	≈11 at gradient 10 μM [46]
Aminoglycoside	Kanamycin	484.50	LamB	*E. coli*	≤1	≤1 at gradient 10 μM [46]
Aminoglycoside	Kanamycin	484.50	Chip	*E. coli*	≤1	≈5 at gradient 10 μM [46]
Aminoglycoside	Amikacin	585.60	OmpF	*E. coli*	≤1	≤1 at gradient 10 μM [46]
Aminoglycoside	Amikacin	585.60	OmpC	*E. coli*	≤1	≤1 at gradient 10 μM [46]
Aminoglycoside	Amikacin	585.60	LamB	*E. coli*	≤1	≤1 at gradient 10 μM [46]
Aminoglycoside	Amikacin	585.60	Chip	*E. coli*	2	≈20 at gradient 10 μM [46]
	Sodium Glutamate Monohydrate	187.12	OprE	*P. aeruginosa*	≤1	≈0.6 at gradient 10 μM [47]
	Arginine	174.20	OprE	*P. aeruginosa*	≤1	≈0.1 at gradient 10 μM [47]

Reduced permeability may results from fewer membrane channels in the outer membrane and their distinct physical and chemical properties. These channels, with their varied structures and specificities, form a diffusional barrier for nonpolar solutes and antibiotic molecules [10,34,39,40,47,49,50,51]. Bacteria may adapt by reducing the influx through these membrane channels, leading to antibiotic resistance [26,33,52,53]. Therefore, studying membrane permeability is crucial for understanding the role of membrane transport in antibiotic resistance mechanisms. Research shows that small polar molecules, typically below 600 Daltons, have the highest activity against Gram-negative bacteria, suggesting that OM-Ps may be the primary entry pathway [10,24,29,32]. However, low antibiotic permeability through the outer membrane may often necessitate high doses of antibiotics, which may lead to toxic side effects [24,32].

The complex processes governing antibiotic uptake and efficacy are multifaceted and shaped by a myriad of bacterial factors. Although the outer membrane of Gram-negative bacteria poses a formidable barrier to antibiotic entry, small hydrophilic molecules can traverse through OM-P pathways [10,24,28,47]. Concurrently, emerging evidence suggests that the cytoplasm and inner membrane possess mechanisms to expel toxic compounds, potentially diminishing the effective dose at the target site [32,33]. Antibiotic resistance may happen in a few ways. For example, bacteria produce enzymes that break down antibiotics, change their outer membrane to block the drugs from entering or alter target proteins inside the cell so the antibiotics cannot work. These different mechanisms make antibiotic resistance a complex issue that requires ongoing research to address [4,54,55,56].

Overcoming bacterial defence mechanisms against antibiotics needs a profound comprehension of the complex processes governing antibiotic uptake, resistance, and delivery mechanisms. There is a pressing need for quantitative data explaining antibiotic uptake dynamics, particularly within various bacterial compartments, as well as innovative strategies to enhance antibiotic delivery and efficacy. The outer membrane of bacteria serves as a critical barrier, significantly influencing the organism’s susceptibility to antibiotics. Emerging evidence highlights the profound impact of structural alterations in outer membrane proteins on antibiotic resistance, especially when coupled with the presence of beta-lactamases in the periplasmic space [10,15,27,32,33,38,56,57]. Unraveling the complex transport mechanisms that underlie membrane permeability is likely to be critical in addressing this challenge, as this knowledge may help in the design of effective antibiotics. Researchers have used a variety of approaches to study a myriad of membrane channels, synthesizing findings to gain deeper insights into the functional role of these channels in outer membrane permeability and their complex interactions with various antibiotics.

The primary objective of this assessment has been build upon the author’s previous work [14,15] by compiling the work on different Om-Ps data and, additionally, the flux rates of different charged antibiotics and compounds through Om-P of Gram-negative bacteria as reported in various studies [40,41,42,43,44,45,46,47]. This review looks to spark discussion on antibiotics and their interactions with outer membrane proteins in Gram-negative bacteria. Understanding these interactions may aid in developing new antibiotics. The challenge of how antibiotics penetrate the cell wall of Gram-negative bacteria is complex. The insights into this process are very crucial for effective antibiotic development, however understanding the molecular basis of outer membrane permeability remains challenging. By combining current knowledge on Om-Ps and their role in antibiotic entry, this review aims to prompt exploration of the factors influencing antibiotic movement through bacterial membranes. This exploration may lead to breakthroughs in combating antibiotic resistance and improving therapeutic strategies.

## 3. Understanding Flux Quantification

**Figure 1:**
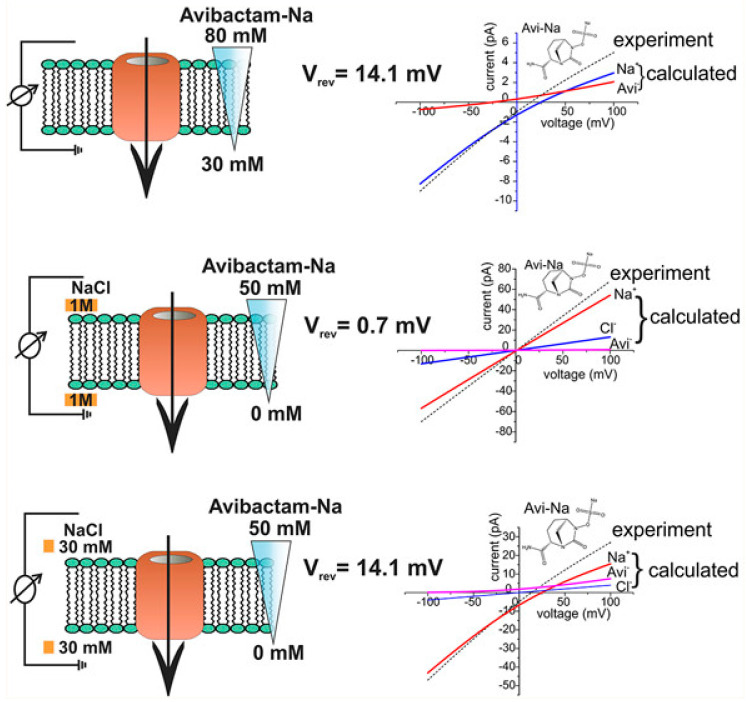
This figure shows a systematic representation of experiments on reversal potential (Vrev) [44]. Adapted with permission from ACS J. Phys. Chem. Lett. 2017, 8, 6, 1295–1301.

The electrophysiological reversal potential assay is a widely used and established method for determining ion flux. It has now been applied innovatively to study the transport of charged antibiotics and compounds through membrane proteins [44,45]. This method involves applying a concentration gradient and measuring the reversal potential, which is the electrostatic potential created by the uneven diffusion of cations and anions across the OM-Ps [44,45]. In this approach, multiple membrane OM-Ps, for example, OmpF and others (see Table 1 for different OM-Ps), are reconstituted into a planar lipid bilayer under symmetric buffer conditions [44,45]. Then, the ion current versus applied voltage is recorded (Figure 1). Under symmetric conditions, zero applied voltage results in no ion current, while under concentration, the gradient generates a reversal potential (Vrev). This difference reflects the electrostatic properties of OM-Ps. For a detailed understanding, please refer to [44,45].

Using the Goldman–Hodgkin–Katz (GHK) ion current equation, the obtained current–voltage (I/V) curves were used to determine the permeability ratio between cations and anions [44,45]. By combining these data with single-channel conductance measurements, a contribution of individual ions to the overall flux can be calculated [41,44,45,46]. The OM-P conductance of a bi-ionic solution of a charged compound further gives the total flux of ions under an external voltage, and the permeability ratio allows to distribute the ion flux between the respective cations and anions [44,45]. However, the concentration-driven flux (see Figure 1) is less obvious as both charges move in the same direction, and only the difference gives rise to an electrical signal [44,45]. This review merges the findings from multiple research studies [40,41,42,43,44,45,46,47,49] that have examined the flux rates of various antibiotics, beta-lactamase inhibitors, and other compounds through the outer membrane proteins of Gram-negative bacteria. The information about these flux rates, as reported across different papers, has been summarised in Table 1. These data offer valuable insights into the permeability characteristics of these membrane proteins and their interactions with different molecular entities. Compiling flux rates in Table 1 serves as a valuable resource for researchers, providing an inclusive reference point for future investigations. 

## 4. Flux Data Analysis

The flux data for specific antibiotics reveal varying permeability profiles across different OM-Ps. For instance, the β-lactamase inhibitor avibactam exhibits a recalculated flux of 62 molecules/s through OmpF and 23 molecules/second through OmpC in *E. coli* at 1 μM concentration, suggesting higher permeability through OmpF [44,45]. In contrast, tazobactam and sulbactam both show a recalculated flux of 20 molecules/s through OmpF and 20 and 18 molecules/s through OmpC, respectively [44,45]. The phosphonic antibiotic fosfomycin displays differential permeability in *P. aeruginosa* and *E. coli* [41,42]. Specifically, fosfomycin shows a recalculated flux of 28 molecules/s through OprO in *P. aeruginosa* and 37 molecules/second through OmpF in *E. coli.* The flux through OmpC and LamB for Fosfomycin in E. coli is significantly lower, at 7 and 3 molecules/s, respectively [41,42].

Cephalosporins, such as ceftazidime, show a flux of 34 molecules/s through OmpF and 17 molecules/s through OmpC [40]. For aminoglycosides like gentamicin, the flux rates are generally low through most OM-Ps, with gentamicin showing 1.5 molecules/s through OmpF and less than 1 molecule/s through OmpC, LamB, and Chip [46].

These findings underscore the variability in antibiotic permeability across different OM-Ps and bacterial species, with OmpF being more permeable than OmpC. Understanding the interactions between antibiotics and OM-Ps can lead to uncovering potential targets for enhancing antibiotic uptake, such as changing antibiotic structures to increase affinity for specific porins. Further research should investigate factors like porin mutations, environmental conditions affecting porin expression, and synergistic effects between antibiotics.

The development of high-throughput screening methods for assessing antibiotic permeability could speed up the discovery of new drugs with improved efficacy against Gram-negative bacteria [24,58]. In summary, this synthesis of current knowledge on the role of OM-Ps in antibiotic translocation highlights the potential for targeting these proteins to combat antibiotic resistance, emphasising the need for continued research in this critical area [24,58].

The results of this study show the complex dynamics that may contribute to membrane transport mechanisms, especially in Gram-negative bacteria. The research underscores the critical role of OM-Ps in mediating antibiotic permeability. Structural alterations in OM-Ps, such as reduced expression, restricted channels, and mutations, may have a significant impact on antibiotic uptake.

## 5. Discussion

The intricate relationship between outer membrane permeability and antibiotic resistance in Gram-negative bacteria underscores a critical challenge in modern medicine [24,26,33]. The selective barrier formed by the outer membrane, primarily through OM-Ps significantly influences antibiotic efficacy [24,26,29,33]. This review highlights the variability in flux rates of different charged antibiotics through various OM-Ps, revealing that even minor structural changes in these proteins can drastically alter antibiotic flux rates.

The data synthesised here illustrate that antibiotics such as avibactam and fosfomycin exhibit varying permeability profiles across different OM-Ps and bacterial species, with OmpF generally showing higher permeability compared to OmpC. This suggests that targeting specific porins may possibly be a viable strategy to enhance antibiotic uptake.

Moreover, this study underscores the need for a deeper understanding of the molecular mechanisms governing antibiotic permeability. Factors such as porin mutations, environmental conditions affecting porin expression, and the presence of efflux pumps add layers of complexity to the issue of antibiotic resistance [10,24,28,59,60]. The electrophysiological reversal potential assay, employed in various studies, has proven effective in quantifying ion flux through these channels, providing valuable insights into the physicochemical properties influencing antibiotic translocation.

Despite the novelty of this review, several limitations should also be acknowledged. This review does not provide a comprehensive comparison with data obtained by other methods, such as molecular dynamics simulations, in vivo studies, or alternative electrophysiological techniques, which could offer a more holistic understanding of antibiotic translocation mechanisms. Additionally, the impact of lipid composition on flux rates is not addressed; the lipid environment surrounding OM-Ps can significantly influence their structure and function, and variations in lipid composition could alter antibiotic permeability. Moreover, possible non-linearity effects of substrate concentration on flux rates are not considered; antibiotic permeability may not always follow a linear relationship with concentration gradients, and potential saturation effects or cooperative interactions between antibiotics and OM-Ps should be investigated. Furthermore, the effect of protein density on the outer membrane, which can influence the overall permeability and function of OM-Ps, has not been studied in detail. Addressing these limitations in future research will enhance our understanding of antibiotic permeability through bacterial outer membranes and improve strategies to combat antibiotic resistance.

Moreover, future research should focus on developing high-throughput screening methods to assess antibiotic permeability across a broader range of OM-Ps. Such advancements could expedite the discovery of new antibiotics with enhanced efficacy against resistant Gram-negative bacteria [10,24,58]. Additionally, exploring synergistic effects between different antibiotics and understanding the role of intracellular mechanisms in antibiotic resistance could pave the way for novel therapeutic strategies.

## 6. Conclusions

Quantitative assessments of antibiotic influx rates through various OM-Ps, including OmpF and OmpC, demonstrate that these channels are pivotal in antibiotic translocation [40,41,43,44,45,46]. The review summarises published data on electrophysiological techniques used to measure ion currents across these channels, offering insights into the electrostatic properties and transport dynamics of antibiotics [44,45]. This approach explains the role of concentration gradients and reversal potentials in antibiotic transport, offering a novel method for characterising the permeability of charged antibiotics [44,45]. The research emphasises the necessity of understanding the physicochemical parameters governing antibiotic translocation. The interplay between antibiotic structure, outer membrane modifications, and beta-lactamases in the periplasmic space is crucial in determining bacterial susceptibility [36,56].

In conclusion, this study’s findings contribute to a deeper understanding of transport across Gram-negative bacterial outer membrane and the role of OM-Ps in antibiotic/molecular flux in Gram-negative bacteria. By highlighting the significance of outer membrane permeability and the factors influencing antibiotic uptake, this research paves the way for the development of more effective therapeutic interventions against resistant bacterial strains. The insights gained from this study may help contribute the design of new antibiotics and the optimisation of existing treatments, to aid in the fight against antibiotic resistance.

## References

[1] Patil P.A., Bobde K.A., Masurkar S.A. (2024). Combating Antimicrobial Resistance: The Role of New Biotechnological Tools. NATURALISTA CAMPANO.

[2] Ahmed S.K., Hussein S., Qurbani K., Ibrahim R.H., Fareeq A., Mahmood K.A., Mohamed M.G. (2024). Antimicrobial resistance: Impacts, challenges, and future prospects. J. Med. Surg. Public Health.

[3] Munita J.M., Arias C.A. (2016). Mechanisms of Antibiotic Resistance. Microbiol Spectr.

[4] Yigit H., Queenan A.M., Anderson G.J., Domenech-Sanchez A., Biddle J.W., Steward C.D., Alberti S., Bush K., Tenover F.C. (2001). Novel carbapenem-hydrolyzing beta-lactamase, KPC-1, from a carbapenem-resistant strain of *Klebsiella pneumoniae*. Antimicrob. Agents Chemother..

[5] Soge O.O., Harger D., Schafer S., Toevs K., Raisler K.A., Venator K., Holmes K.K., Kirkcaldy R.D. (2012). Emergence of increased azithromycin resistance during unsuccessful treatment of *Neisseria gonorrhoeae* infection with azithromycin (Portland, OR, 2011). Sex. Transm. Dis..

[6] Mangili A., Bica I., Snydman D.R., Hamer D.H. (2005). Daptomycin-resistant, methicillin-resistant *Staphylococcus aureus* bacteremia. Clin. Infect. Dis..

[7] Jevons M.P., Rolinson G.N., Knox R. (1961). Celbenin-Resistant Staphylococci. Br. Med. J..

[8] Humphries R.M., Yang S., Hemarajata P., Ward K.W., Hindler J.A., Miller S.A., Gregson A. (2015). First Report of Ceftazidime-Avibactam Resistance in a KPC-3-Expressing *Klebsiella pneumoniae* Isolate. Antimicrob. Agents Chemother..

[9] Ventola C.L. (2015). The antibiotic resistance crisis: Part 1: Causes and threats. Pharm. Ther..

[10] Masi M., Winterhalter M., Pages J.M. (2019). Outer Membrane Porins. Bacterial Cell Walls and Membranes.

[11] Larsson D.G.J., Flach C.F. (2022). Antibiotic resistance in the environment. Nat. Rev. Microbiol..

[12] Muteeb G., Rehman M.T., Shahwan M., Aatif M. (2023). Origin of antibiotics and antibiotic resistance, and their impacts on drug development: A narrative review. Pharmaceuticals.

[13] Klug D.M., Idiris F.I., Blaskovich M.A., von Delft F., Dowson C.G., Kirchhelle C., Roberts A.P., Singer A.C., Todd M.H. (2021). There is no market for new antibiotics: This allows an open approach to research and development. Wellcome Open Res..

[14] Ghai I. (2023). A Barrier to Entry: Examining the Bacterial Outer Membrane and Antibiotic Resistance. Appl. Sci..

[15] Ghai I., Ghai S. (2017). Exploring bacterial outer membrane barrier to combat bad bugs. Infect. Drug Resist..

[16] Petrova E. (2013). Innovation in the pharmaceutical industry: The process of drug discovery and development. Innovation and Marketing in the Pharmaceutical Industry: Emerging Practices, Research, and Policies.

[17] Shedeed E. (2024). Mapping Global Governance of Antibiotic Stewardship: A One Health Multi-Level Governance Approach. Ph.D. Thesis.

[18] Bartfai T., Lees G.V. (2013). The Future of Drug Discovery: Who Decides Which Diseases To Treat?.

[19] Cordell G.A. (2024). The contemporary nexus of medicines security and bioprospecting: A future perspective for prioritizing the patient. Nat. Prod. Bioprospect..

[20] Sharma S., Chauhan A., Ranjan A., Mathkor D.M., Haque S., Ramniwas S., Tuli H.S., Jindal T., Yadav V. (2024). Emerging challenges in antimicrobial resistance: Implications for pathogenic microorganisms, novel antibiotics, and their impact on sustainability. Front. Microbiol..

[21] Soni J., Sinha S., Pandey R. (2024). Understanding bacterial pathogenicity: A closer look at the journey of harmful microbes. Front. Microbiol..

[22] Ahmed F., Shamim N.J., Das A., Sharma H.K., Grewal A.S., Pandita D., Lather V. (2024). Combating antimicrobial resistance: A paradigm shift from general to precision medicine. Chem. Biol. Lett..

[23] Roque-Borda C.A., Primo L.M.D.G., Franzyk H., Hansen P.R., Pavan F.R. (2024). Recent Advances in the Development of Antimicrobial Peptides against ESKAPE Pathogens. Heliyon.

[24] Winterhalter M. (2021). Antibiotic uptake through porins located in the outer membrane of Gram-negative bacteria. Expert Opin. Drug Deliv..

[25] Oliveira J., Reygaert W.C. (2022). Gram Negative Bacteria. StatPearls.

[26] Nikaido H., Nakae T. (1979). The outer membrane of Gram-negative bacteria. Adv. Microb. Physiol..

[27] Delcour A.H. (2009). Outer membrane permeability and antibiotic resistance. Biochim. Biophys. Acta.

[28] Pages J.M., James C.E., Winterhalter M. (2008). The porin and the permeating antibiotic: A selective diffusion barrier in Gram-negative bacteria. Nat. Rev. Microbiol..

[29] Winterhalter M., Ceccarelli M. (2015). Physical methods to quantify small antibiotic molecules uptake into Gram-negative bacteria. Eur. J. Pharm. Biopharm..

[30] Modi N., Benz R., Hancock R.E., Kleinekathofer U. (2012). Modeling the Ion Selectivity of the Phosphate Specific Channel OprP. J. Phys. Chem. Lett..

[31] Alcaraz A., Nestorovich E.M., Lopez M.L., Garcia-Gimenez E., Bezrukov S.M., Aguilella V.M. (2009). Diffusion, exclusion, and specific binding in a large channel: A study of OmpF selectivity inversion. Biophys. J..

[32] Weingart H., Petrescu M., Winterhalter M. (2008). Biophysical characterization of in- and efflux in Gram-negative bacteria. Curr. Drug Targets.

[33] Nikaido H. (1994). Prevention of drug access to bacterial targets: Permeability barriers and active efflux. Science.

[34] Masi M., Pages J.M. (2013). Structure, Function and Regulation of Outer Membrane Proteins Involved in Drug Transport in Enterobactericeae: The OmpF/C—TolC Case. Open Microbiol. J..

[35] Ceccarelli M., Danelon C., Laio A., Parrinello M. (2004). Microscopic Mechanism of Antibiotics Translocation through a Porin. Biophys. J..

[36] Pages J.M., Peslier S., Keating T.A., Lavigne J.P., Nichols W.W. (2015). Role of the Outer Membrane and Porins in Susceptibility of beta-Lactamase-Producing Enterobacteriaceae to Ceftazidime-Avibactam. Antimicrob. Agents Chemother..

[37] James C.E., Mahendran K.R., Molitor A., Bolla J.M., Bessonov A.N., Winterhalter M., Pages J.M. (2009). How beta-lactam antibiotics enter bacteria: A dialogue with the porins. PLoS ONE.

[38] Bajaj H., Scorciapino M.A., Moynie L., Page M.G., Naismith J.H., Ceccarelli M., Winterhalter M. (2016). Molecular Basis of Filtering Carbapenems by Porins from beta-Lactam-resistant Clinical Strains of *Escherichia coli*. J. Biol. Chem..

[39] Acosta-Gutierrez S., Ferrara L., Pathania M., Masi M., Wang J., Bodrenko I., Zahn M., Winterhalter M., Stavenger R.A., Pages J.M. (2018). Getting Drugs into Gram-Negative Bacteria: Rational Rules for Permeation through General Porins. ACS Infect. Dis..

[40] Masi M., Vergalli J., Ghai I., Barba-Bon A., Schembri T., Nau W.M., Lafitte D., Winterhalter M., Pages J.M. (2022). Cephalosporin translocation across enterobacterial OmpF and OmpC channels, a filter across the outer membrane. Commun. Biol..

[41] Bianchi M., Winterhalter M., Harbig T.A., Hörömpöli D., Ghai I., Nieselt K., Brötz-Oesterhelt H., Mayer C., Borisova-Mayer M. (2024). Fosfomycin Uptake in *Escherichia coli* Is Mediated by the Outer-Membrane Porins OmpF, OmpC, and LamB. ACS Infect. Dis..

[42] Citak F., Ghai I., Rosenkotter F., Benier L., Winterhalter M., Wagner R. (2018). Probing transport of fosfomycin through substrate specific OprO and OprP from *Pseudomonas aeruginosa*. Biochem. Biophys. Res. Commun..

[43] Ghai I., Bajaj H., Arun Bafna J., El Damrany Hussein H.A., Winterhalter M., Wagner R. (2018). Ampicillin permeation across OmpF, the major outer-membrane channel in *Escherichia coli*. J. Biol. Chem..

[44] Ghai I., Pira A., Scorciapino M.A., Bodrenko I., Benier L., Ceccarelli M., Winterhalter M., Wagner R. (2017). General Method to Determine the Flux of Charged Molecules through Nanopores Applied to beta-Lactamase Inhibitors and OmpF. J. Phys. Chem. Lett..

[45] Ghai I., Winterhalter M., Wagner R. (2017). Probing transport of charged beta-lactamase inhibitors through OmpC, a membrane channel from *E. coli*. Biochem. Biophys. Res. Commun..

[46] Paul E., Ghai I., Hörömpöli D., Brötz-Oesterhelt H., Winterhalter M., Bafna J.A. (2022). Uptake of aminoglycosides through outer membrane porins in *Escherichia coli*. bioRxiv.

[47] Samanta S., D’Agostino T., Ghai I., Pathania M., Acosta Gutierrez S., Andrea Scorciapino M., Bodrenko I., Wagner R., van den Berg B., Winterhalter M. (2017). How to Get Large Drugs through Small Pores? Exploiting the Porins Pathway in *Pseudomonas aeruginosa*. Biophys. J..

[48] Bhamidimarri S.P., Zahn M., Prajapati J.D., Schleberger C., Söderholm S., Hoover J., West J., Kleinekathöfer U., Bumann D., Winterhalter M. (2019). A Multidisciplinary Approach toward Identification of Antibiotic Scaffolds for *Acinetobacter baumannii*. Structure.

[49] Samanta S., Bodrenko I., Acosta-Gutierrez S., D’Agostino T., Pathania M., Ghai I., Schleberger C., Bumann D., Wagner R., Winterhalter M. (2018). Getting Drugs through Small Pores: Exploiting the Porins Pathway in *Pseudomonas aeruginosa*. ACS Infect. Dis..

[50] Acosta Gutierrez S., Bodrenko I., Scorciapino M.A., Ceccarelli M. (2016). Macroscopic electric field inside water-filled biological nanopores. Phys. Chem. Chem. Phys. PCCP.

[51] Bodrenko I.V., Wang J., Salis S., Winterhalter M., Ceccarelli M. (2017). Sensing Single Molecule Penetration into Nanopores: Pushing the Time Resolution to the Diffusion Limit. ACS Sens..

[52] Nestorovich E.M., Sugawara E., Nikaido H., Bezrukov S.M. (2006). *Pseudomonas aeruginosa* porin OprF: Properties of the channel. J. Biol. Chem..

[53] Sugawara E., Kojima S., Nikaido H. (2016). *Klebsiella pneumoniae* Major Porins OmpK35 and OmpK36 Allow More Efficient Diffusion of beta-Lactams than Their *Escherichia coli* Homologs OmpF and OmpC. J. Bacteriol..

[54] Castanheira M., Mendes R.E., Sader H.S. (2017). Low Frequency of Ceftazidime-Avibactam Resistance among *Enterobacteriaceae* Isolates Carrying *bla*_KPC_ Collected in U.S. Hospitals from 2012 to 2015. Antimicrob. Agents Chemother..

[55] Bornet C., Davin-Regli A., Bosi C., Pages J.M., Bollet C. (2000). Imipenem resistance of enterobacter aerogenes mediated by outer membrane permeability. J. Clin. Microbiol..

[56] Dever L.A., Dermody T.S. (1991). Mechanisms of bacterial resistance to antibiotics. Arch. Intern. Med..

[57] Bajaj H., Tran Q.T., Mahendran K.R., Nasrallah C., Colletier J.P., Davin-Regli A., Bolla J.M., Pages J.M., Winterhalter M. (2012). Antibiotic uptake through membrane channels: Role of *Providencia stuartii* OmpPst1 porin in carbapenem resistance. Biochemistry.

[58] Stavenger R.A., Winterhalter M. (2014). TRANSLOCATION project: How to get good drugs into bad bugs. Sci. Transl. Med..

[59] Liu N., Samartzidou H., Lee K.W., Briggs J.M., Delcour A.H. (2000). Effects of pore mutations and permeant ion concentration on the spontaneous gating activity of OmpC porin. Protein Eng..

[60] Danelon C., Suenaga A., Winterhalter M., Yamato I. (2003). Molecular origin of the cation selectivity in OmpF porin: Single channel conductances vs. free energy calculation. Biophys. Chem..

